# Supplementary material for: A Likelihood Approach for Real-Time Calibration of Stochastic Compartmental Epidemic Models
Source: PLoS Comput Biol. 2017 Jan 17;13(1):e1005257. doi: 10.1371/journal.pcbi.1005257 (PMC5240920; doi:10.1371/journal.pcbi.1005257)
Supplement: S1 File — (TAR.GZ) [file pcbi.1005257.s014.tar.gz › HSPH_Online-SI-Revision/MSS10/n100-extreme/n100-extreme_NEW_table8.pdf]

| | | | | | | | | | | | | | | | | | | | | | | | | | | | |
|---|---|---|---|---|---|---|---|---|---|---|---|---|---|---|---|---|---|---|---|---|---|---|---|---|---|---|---|
| 1 | R0 | {2.76052, 16.3913, 0.1} | {2.93371, 17.1903, 1.} | {{2.93371, 17.1903, 1.}, {2.93371, 17.1903, 1.}} | {6.27367, 4.87452, 900.} | inf | 3200. | 3139.09 | {3114.36, 3161.15} | 1.90356 | 0.00835529 | newly | 1155. | 1099.97 | {1066., 1135.} | 4.85281 | 0.0217213 | Reff | 0.245824 | 0.263034 | {0.262208, 0.263743} | 7.00063 | ar | 92 090. | 92 066.3 | {92 005., 92 127.} | 0.0465414 |
| 2 | R0 | {2.18202, 15.1734, 3.} | {2.24606, 16.668, 3.} | {{2.24606, 16.668, 3.}, {2.24606, 16.668, 3.}} | {2.9351, 9.85011, 0.} | inf | 3327. | 3458.15 | {3422.56, 3491.14} | 3.94209 | 0.0167805 | newly | 1337. | 1283.62 | {1240., 1319.} | 4.05385 | 0.0180686 | Reff | 0.385257 | 0.393619 | {0.392868, 0.394413} | 2.17047 | ar | 84 264. | 84 494.1 | {84 393., 84 597.} | 0.273664 |
| 3 | R0 | {2.57891, 12.5971, 1.} | {2.5898, 12.9574, 2.} | {{2.5898, 12.9574, 2.}, {2.5898, 12.9574, 2.}} | {0.422351, 2.86046, 100.} | inf | 1362. | 1304.36 | {1284.14, 1321.09} | 4.23188 | 0.0188081 | newly | 609. | 577.78 | {554., 606.} | 5.23481 | 0.0235672 | Reff | 0.262765 | 0.265368 | {0.264878, 0.265866} | 0.990432 | ar | 90 302. | 90 209.2 | {90 167., 90 256.} | 0.103619 |
| 4 | R0 | {2.5856, 9.28508, 0.1} | {2.59708, 9.39342, 0.} | {{2.59708, 9.39342, 0.}, {2.59708, 9.39342, 0.}} | {0.44405, 1.16682, 100.} | inf | 433. | 286.746 | {273.956, 300.014} | 33.777 | 0.179233 | newly | 254. | 162.71 | {149., 177.} | 35.9409 | 0.194307 | Reff | 0.254526 | 0.259455 | {0.259097, 0.259778} | 1.93637 | ar | 90 312. | 90 108.9 | {90 087., 90 128.} | 0.224854 |
| 5 | R0 | {2.4406, 12.1341, 1.} | {2.44101, 12.0941, 0.} | {{2.44101, 12.0941, 0.}, {2.44101, 12.0941, 0.}} | {0.0168666, 0.3296, 100.} | inf | 1891. | 1435.41 | {1413.61, 1455.75} | 24.0926 | 0.119746 | newly | 894. | 683.73 | {655., 711.} | 23.5201 | 0.116682 | Reff | 0.297534 | 0.308705 | {0.308184, 0.30921} | 3.75461 | ar | 88 597. | 87 970.9 | {87 914., 88 026.} | 0.706649 |
| 6 | R0 | {2.72155, 15.4094, 2.} | {2.7499, 15.3124, 0.} | {{2.7499, 15.3124, 0.}, {2.7499, 15.3124, 0.}} | {1.04159, 0.629402, 100.} | inf | 3044. | 3131.01 | {3105.75, 3160.8} | 2.8583 | 0.0122275 | newly | 1132. | 1210.69 | {1165., 1251.} | 6.97615 | 0.0291246 | Reff | 0.252533 | 0.25277 | {0.251839, 0.253456} | 0.216507 | ar | 91 682. | 91 789.6 | {91 727., 91 849.} | 0.117493 |
| 7 | R0 | {2.63781, 14.3935, 0.1} | {2.96797, 18.4042, 0.} | {{2.96797, 18.4042, 0.}, {2.96797, 18.4042, 0.}} | {12.5165, 27.8647, 100.} | inf | 2374. | 4066.43 | {4043.58, 4092.86} | 71.2903 | 0.233727 | newly | 968. | 1339.45 | {1303., 1381.} | 38.3729 | 0.14094 | Reff | 0.252043 | 0.233359 | {0.232564, 0.234021} | 7.41296 | ar | 91 192. | 93 259.6 | {93 201., 93 325.} | 2.26726 |
| 8 | R0 | {2.82391, 16.104, 1.} | {2.93371, 17.1903, 1.} | {{2.93371, 17.1903, 1.}, {2.93371, 17.1903, 1.}} | {3.88808, 6.74551, 0.} | inf | 3239. | 3936.02 | {3907.52, 3962.27} | 21.5195 | 0.0846389 | newly | 1178. | 1376.22 | {1327., 1417.} | 16.8268 | 0.067405 | Reff | 0.232408 | 0.220996 | {0.220197, 0.221807} | 4.9104 | ar | 92 664. | 93 483.3 | {93 422., 93 530.} | 0.884151 |
| 9 | R0 | {2.80645, 14.6457, 0.1} | {2.5684, 13.7324, 1.} | {{2.5684, 13.7324, 1.}, {2.5684, 13.7324, 1.}} | {8.48224, 6.23572, 900.} | inf | 2875. | 2540.76 | {2509.96, 2563.63} | 11.6258 | 0.053687 | newly | 1160. | 1068.72 | {1035., 1099.} | 7.86897 | 0.035699 | Reff | 0.229399 | 0.218526 | {0.217887, 0.219242} | 4.74001 | ar | 92 567. | 92 162.7 | {92 110., 92 225.} | 0.436732 |
| 10 | R0 | {1.86213, 5.4271, 0.1} | {1.83544, 5.78475, 5.} | {{1.83544, 5.78475, 5.}, {1.83544, 5.78475, 5.}} | {1.43347, 6.59003, 4900.} | inf | 95. | 68.6252 | {53.0497, 87.6556} | 27.7692 | 0.148644 | newly | 92. | 60.86 | {44., 77.} | 33.8478 | 0.187986 | Reff | 0.460598 | 0.454479 | {0.454127, 0.454746} | 1.32837 | ar | 75 362. | 75 297.5 | {75 259., 75 340.} | 0.087365 |
| 11 | R0 | {2.27349, 14.6338, 1.} | {2.40085, 15.146, 2.} | {{2.40085, 15.146, 2.}, {2.40085, 15.146, 2.}} | {5.60197, 3.50004, 100.} | inf | 2757. | 4379.66 | {4340.69, 4414.17} | 58.8559 | 0.200994 | newly | 1137. | 1741.08 | {1698., 1777.} | 53.1293 | 0.184988 | Reff | 0.351982 | 0.332742 | {0.331891, 0.333624} | 5.4661 | ar | 85 805. | 88 085.5 | {87 990., 88 169.} | 2.65776 |
| 12 | R0 | {2.77261, 14.1894, 0.1} | {2.7499, 15.3124, 0.} | {{2.7499, 15.3124, 0.}, {2.7499, 15.3124, 0.}} | {0.819182, 7.91445, 100.} | inf | 2393. | 2120.17 | {2100.98, 2140.21} | 11.4011 | 0.0525844 | newly | 959. | 815.17 | {783., 844.} | 14.9979 | 0.0707545 | Reff | 0.226855 | 0.232499 | {0.23193, 0.23301} | 2.488 | ar | 92 508. | 92 161.1 | {92 115., 92 201.} | 0.374995 |
| 13 | R0 | {2.65402, 19.3935, 2.} | {2.30907, 14.0824, 0.} | {{2.30907, 14.0824, 0.}, {2.30907, 14.0824, 0.}} | {12.9972, 27.3859, 100.} | inf | 6091. | 3622.89 | {3581.68, 3661.2} | 40.5205 | 0.225647 | newly | 2008. | 1525.88 | {1491., 1561.} | 24.01 | 0.119329 | Reff | 0.292659 | 0.311612 | {0.31072, 0.31252} | 6.47609 | ar | 91 184. | 88 005.4 | {87 907., 88 086.} | 3.48597 |
| 14 | R0 | {1.75297, 4.45354, 0.1} | {1.81179, 4.06746, 2.} | {{1.81179, 4.06746, 2.}, {1.81179, 4.06746, 2.}} | {3.35535, 8.66915, 1900.} | inf | 33. | 37.7863 | {23.1051, 52.5846} | 29.7402 | 0.122178 | newly | 37. | 44.6 | {27., 64.} | 34.4865 | 0.134587 | Reff | 0.50468 | 0.521527 | {0.521257, 0.52179} | 3.33817 | ar | 71 263. | 71 254.6 | {71 218., 71 304.} | 0.0338605 |
| 15 | R0 | {1.99928, 7.1501, 0.1} | {1.9862, 6.28417, 0.} | {{1.9862, 6.28417, 0.}, {1.9862, 6.28417, 0.}} | {0.654243, 12.1108, 100.} | inf | 273. | 7.22559 | {2.25273, 12.7948} | 97.3533 | 1.66993 | newly | 197. | 5.84 | {2., 11.} | 97.0355 | 1.6666 | Reff | 0.411872 | 0.414456 | {0.414339, 0.414554} | 0.627421 | ar | 79 602. | 79 139.1 | {79 129., 79 152.} | 0.581568 |
| 16 | R0 | {2.67398, 10.3134, 1.} | {2.68409, 10.6354, 4.} | {{2.68409, 10.6354, 4.}, {2.68409, 10.6354, 4.}} | {0.378091, 3.12193, 300.} | inf | 624. | 655.841 | {640.434, 670.224} | 5.10274 | 0.0215458 | newly | 342. | 339.47 | {316., 358.} | 3.7924 | 0.0166275 | Reff | 0.237957 | 0.238003 | {0.237612, 0.238413} | 0.112269 | ar | 91 289. | 91 335.5 | {91 300., 91 367.} | 0.0517587 |
| 17 | R0 | {2.44634, 16.3226, 0.1} | {2.24606, 16.668, 3.} | {{2.24606, 16.668, 3.}, {2.24606, 16.668, 3.}} | {8.18674, 2.11606, 2900.} | inf | 3997. | 3161.65 | {3128.46, 3194.9} | 20.8994 | 0.101834 | newly | 1461. | 1147.23 | {1118., 1181.} | 21.4764 | 0.105104 | Reff | 0.317486 | 0.310257 | {0.309473, 0.31098} | 2.27704 | ar | 88 710. | 87 508.1 | {87 429., 87 597.} | 1.3549 |
| 18 | R0 | {2.02168, 10.1676, 0.1} | {2.21411, 14.2212, 2.} | {{2.21411, 14.2212, 2.}, {2.21411, 14.2212, 2.}} | {9.51822, 39.8678, 1900.} | inf | 1489. | 2482.83 | {2458.22, 2508.76} | 66.7447 | 0.222035 | newly | 847. | 1068.9 | {1036., 1104.} | 26.1983 | 0.100912 | Reff | 0.420772 | 0.438818 | {0.438241, 0.439357} | 4.28869 | ar | 80 299. | 81 955.4 | {81 840., 82 061.} | 2.0628 |
| 19 | R0 | {2.89969, 15.009, 0.1} | {2.96947, 15.963, 0.} | {{2.96947, 15.963, 0.}, {2.96947, 15.963, 0.}} | {2.40652, 6.35593, 100.} | inf | 2686. | 4041.91 | {4015.77, 4064.11} | 50.4807 | 0.177477 | newly | 1026. | 1490.26 | {1451., 1527.} | 45.2495 | 0.162018 | Reff | 0.211938 | 0.176775 | {0.17611, 0.177445} | 16.5912 | ar | 93 349. | 94 841.8 | {94 788., 94 891.} | 1.59915 |
| 20 | R0 | {2.68933, 7.34928, 0.1} | {2.72208, 7.16132, 4.} | {{2.72208, 7.16132, 4.}, {2.72208, 7.16132, 4.}} | {1.2178, 2.55758, 3900.} | inf | 136. | 631.209 | {615.79, 645.551} | 364.124 | 0.666563 | newly | 80. | 430.8 | {410., 449.} | 438.5 | 0.730911 | Reff | 0.227975 | 0.217271 | {0.216876, 0.217688} | 4.69513 | ar | 91 574. | 92 190.6 | {92 154., 92 222.} | 0.673302 |
| 21 | R0 | {2.15352, 9.28074, 0.1} | {2.32891, 10.9956, 0.} | {{2.32891, 10.9956, 0.}, {2.32891, 10.9956, 0.}} | {8.1442, 18.4772, 100.} | inf | 838. | 814.946 | {794.891, 837.11} | 2.91597 | 0.0129131 | newly | 504. | 426.06 | {403., 446.} | 15.4643 | 0.0732843 | Reff | 0.358733 | 0.388486 | {0.387962, 0.38893} | 8.29387 | ar | 83 845. | 83 823.7 | {83 762., 83 879.} | 0.0496392 |
| 22 | R0 | {2.21953, 9.04387, 0.1} | {2.32678, 10.0266, 4.} | {{2.32678, 10.0266, 4.}, {2.32678, 10.0266, 4.}} | {4.83231, 10.8667, 3900.} | inf | 694. | 2288.3 | {2254.26, 2317.19} | 229.726 | 0.51813 | newly | 414. | 1267.7 | {1232., 1304.} | 206.208 | 0.485908 | Reff | 0.343938 | 0.323463 | {0.322642, 0.324176} | 5.9533 | ar | 84 856. | 87 134.5 | {87 048., 87 228.} | 2.68509 |
| 23 | R0 | {2.21987, 14.5347, 1.} | {2.35866, 16.9062, 2.} | {{2.35866, 16.9062, 2.}, {2.35866, 16.9062, 2.}} | {6.25225, 16.3163, 100.} | inf | 2885. | 3078.48 | {3042.64, 3110.57} | 6.70658 | 0.028174 | newly | 1192. | 1123.48 | {1090., 1155.} | 5.77685 | 0.0259697 | Reff | 0.366057 | 0.38438 | {0.383607, 0.38521} | 5.00554 | ar | 85 067. | 85 455.7 | {85 368., 85 530.} | 0.456875 |
| 24 | R0 | {2.84268, 17.9659, 1.} | {2.76795, 19.6041, 1.} | {{2.76795, 19.6041, 1.}, {2.76795, 19.6041, 1.}} | {2.62889, 9.11863, 0.} | inf | 4541. | 7727.58 | {7696.37, 7751.89} | 70.1736 | 0.23089 | newly | 1477. | 2370.75 | {2319., 2425.} | 60.5112 | 0.205449 | Reff | 0.242139 | 0.147571 | {0.146782, 0.148322} | 39.0554 | ar | 92 836. | 95 831.1 | {95 785., 95 877.} | 3.22626 |
| 25 | R0 | {2.52706, 10.3679, 0.1} | {2.42049, 9.29056, 4.} | {{2.42049, 9.29056, 4.}, {2.42049, 9.29056, 4.}} | {4.21735, 10.3911, 3900.} | inf | 746. | 948.191 | {926.486, 969.088} | 27.1034 | 0.104098 | newly | 432. | 543.8 | {519., 569.} | 25.8796 | 0.0996778 | Reff | 0.269107 | 0.252863 | {0.252357, 0.253388} | 6.03596 | ar | 89 623. | 89 865.3 | {89 817., 89 908.} | 0.270399 |
| 26 | R0 | {2.62835, 19.1088, 1.} | {2.29055, 13.7413, 0.} | {{2.29055, 13.7413, 0.}, {2.29055, 13.7413, 0.}} | {12.8522, 28.0891, 100.} | inf | 5501. | 2167.86 | {2141.52, 2194.39} | 60.5916 | 0.404433 | newly | 1758. | 936.63 | {906., 963.} | 46.7218 | 0.273578 | Reff | 0.292378 | 0.331148 | {0.330479, 0.331749} | 13.2604 | ar | 90 908. | 86 551.4 | {86 477., 86 618.} | 4.79232 |
| 27 | R0 | {2.76831, 19.4155, 2.} | {2.86656, 18.9813, 2.} | {{2.86656, 18.9813, 2.}, {2.86656, 18.9813, 2.}} | {3.54904, 2.23611, 0.} | inf | 4502. | 5714.39 | {5685.39, 5739.59} | 26.9301 | 0.103561 | newly | 1418. | 1834.25 | {1791., 1879.} | 29.3547 | 0.111709 | Reff | 0.252857 | 0.227078 | {0.226285, 0.227892} | 10.1954 | ar | 92 175. | 93 543.1 | {93 485., 93 602.} | 1.48423 |
| 28 | R0 | {2.96609, 16.8903, 1.} | {2.63407, 13.6136, 3.} | {{2.63407, 13.6136, 3.}, {2.63407, 13.6136, 3.}} | {11.1938, 19.4002, 200.} | inf | 3132. | 1607.13 | {1583.69, 1628.5} | 48.6868 | 0.289799 | newly | 1123. | 679.03 | {652., 703.} | 39.5343 | 0.21867 | Reff | 0.198639 | 0.21657 | {0.216007, 0.217167} | 9.02686 | ar | 94 024. | 92 208.1 | {92 162., 92 254.} | 1.93127 |
| 29 | R0 | {2.17046, 14.39, 0.1} | {2.11292, 12.9719, 0.} | {{2.11292, 12.9719, 0.}, {2.11292, 12.9719, 0.}} | {2.65108, 9.85466, 100.} | inf | 3337. | 2100.52 | {2063.89, 2135.54} | 37.0536 | 0.201069 | newly | 1397. | 968.25 | {932., 1000.} | 30.6908 | 0.159377 | Reff | 0.390705 | 0.406472 | {0.405702, 0.407238} | 4.03576 | ar | 84 001. | 82 102.9 | {82 002., 82 193.} | 2.25962 |
| 30 | R0 | {2.88772, 12.4647, 1.} | {2.93502, 12.6864, 3.} | {{2.93502, 12.6864, 3.}, {2.93502, 12.6864, 3.}} | {1.63792, 1.77857, 200.} | inf | 1402. | 1280.77 | {1260.4, 1300.24} | 8.6467 | 0.0393012 | newly | 651. | 571.31 | {548., 593.} | 12.2412 | 0.0569171 | Reff | 0.203498 | 0.210389 | {0.209814, 0.210978} | 3.38636 | ar | 93 273. | 93 157.5 | {93 113., 93 195.} | 0.123787 |
| 31 | R0 | {2.46831, 8.14308, 0.1} | {2.59708, 9.39342, 0.} | {{2.59708, 9.39342, 0.}, {2.59708, 9.39342, 0.}} | {5.21698, 15.3546, 100.} | inf | 300. | 946.33 | {927.742, 962.218} | 215.443 | 0.49887 | newly | 189. | 543.43 | {515., 570.} | 187.529 | 0.458399 | Reff | 0.280894 | 0.278762 | {0.278326, 0.279235} | 0.758837 | ar | 88 762. | 89 628.1 | {89 586., 89 674.} | 0.9758 |
| 32 | R0 | {1.93236, 7.38987, 0.1} | {2.0548, 9.05427, 2.} | {{2.0548, 9.05427, 2.}, {2.0548, 9.05427, 2.}} | {6.33651, 22.5228, 1900.} | inf | 389. | 1357.73 | {1326.96, 1385.9} | 249.03 | 0.542803 | newly | 288. | 851.47 | {820., 884.} | 195.649 | 0.470579 | Reff | 0.437467 | 0.445282 | {0.444634, 0.445902} | 1.78636 | ar | 77 654. | 79 376.2 | {79 290., 79 477.} | 2.21774 |
| 33 | R0 | {2.82251, 17.6954, 1.} | {2.77502, 16.515, 2.} | {{2.77502, 16.515, 2.}, {2.77502, 16.515, 2.}} | {1.68241, 6.67053, 100.} | inf | 4866. | 3273.26 | {3245.64, 3301.26} | 32.732 | 0.172201 | newly | 1670. | 1200.18 | {1161., 1235.} | 28.1329 | 0.143592 | Reff | 0.244147 | 0.284238 | {0.283419, 0.284986} | 16.421 | ar | 92 745. | 90 947.6 | {90 864., 91 020.} | 1.93795 |
| 34 | R0 | {1.87083, 12.3994, 1.} | {2.14288, 16.6365, 3.} | {{2.14288, 16.6365, 3.}, {2.14288, 16.6365, 3.}} | {14.5417, 34.1715, 200.} | inf | 3095. | 5157.89 | {5100.85, 5212.05} | 66.6524 | 0.221798 | newly | 1528. | 1961.39 | {1915., 2009.} | 28.3632 | 0.108374 | Reff | 0.498595 | 0.526894 | {0.525721, 0.528041} | 5.67574 | ar | 76 282. | 80 082.4 | {79 916., 80 235.} | 4.98207 |
| 35 | R0 | {2.95779, 16.3003, 1.} | {2.96947, 15.963, 0.} | {{2.96947, 15.963, 0.}, {2.96947, 15.963, 0.}} | {0.394945, 2.06952, 100.} | inf | 3818. | 4541.26 | {4516.3, 4565.8} | 18.9435 | 0.0753363 | newly | 1428. | 1680.57 | {1630., 1720.} | 17.687 | 0.0706441 | Reff | 0.209086 | 0.188435 | {0.187689, 0.189164} | 9.87696 | ar | 93 873. | 94 606.6 | {94 550., 94 662.} | 0.781503 |
| 36 | R0 | {2.18718, 14.261, 3.} | {2.42074, 15.7308, 3.} | {{2.42074, 15.7308, 3.}, {2.42074, 15.7308, 3.}} | {10.6788, 10.3064, 0.} | inf | 3033. | 5285.82 | {5243.96, 5322.02} | 74.2771 | 0.241234 | newly | 1295. | 2048.51 | {2000., 2099.} | 58.1861 | 0.199106 | Reff | 0.381269 | 0.367449 | {0.366571, 0.36844} | 3.62477 | ar | 84 379. | 87 462.7 | {87 337., 87 567.} | 3.65461 |
| 37 | R0 | {2.44912, 9.31118, 0.1} | {2.42049, 9.29056, 4.} | {{2.42049, 9.29056, 4.}, {2.42049, 9.29056, 4.}} | {1.16919, 0.221464, 3900.} | inf | 559. | 172.627 | {155.184, 186.206} | 69.1186 | 0.511324 | newly | 306. | 101.11 | {89., 116.} | 66.9575 | 0.482904 | Reff | 0.289559 | 0.295526 | {0.295197, 0.295944} | 2.06058 | ar | 88 442. | 87 861.9 | {87 830., 87 891.} | 0.65591 |
| 38 | R0 | {2.05949, 5.72514, 0.1} | {2.0598, 5.9936, 4.} | {{2.0598, 5.9936, 4.}, {2.0598, 5.9936, 4.}} | {0.015208, 4.6892, 3900.} | inf | 81. | 29.8425 | {20.2687, 39.36} | 63.1573 | 0.448796 | newly | 76. | 24.72 | {15., 34.} | 67.4737 | 0.509157 | Reff | 0.380141 | 0.381252 | {0.381052, 0.381436} | 0.292406 | ar | 81 587. | 81 508.7 | {81 489., 81 529.} | 0.0959467 |
| 39 | R0 | {1.75505, 8.52404, 0.1} | {1.77826, 9.06807, 1.} | {{1.77826, 9.06807, 1.}, {1.77826, 9.06807, 1.}} | {1.32228, 6.38229, 900.} | inf | 1029. | 563.035 | {529.551, 597.665} | 45.2833 | 0.262349 | newly | 719. | 364.31 | {334., 395.} | 49.331 | 0.296091 | Reff | 0.527357 | 0.542617 | {0.541973, 0.5432} | 2.89352 | ar | 70 925. | 70 135.3 | {70 018., 70 248.} | 1.1134 |
| 40 | R0 | {2.24724, 13.7861, 1.} | {2.18932, 13.5121, 4.} | {{2.18932, 13.5121, 4.}, {2.18932, 13.5121, 4.}} | {2.57746, 1.9875, 300.} | inf | 2420. | 3957.98 | {3925.17, 3989.88} | 63.5528 | 0.213649 | newly | 1045. | 1726.57 | {1683., 1770.} | 65.222 | 0.21798 | Reff | 0.360008 | 0.317058 | {0.316341, 0.317765} | 11.9304 | ar | 85 257. | 87 195.3 | {87 109., 87 274.} | 2.27346 |
| 41 | R0 | {1.77635, 8.62406, 0.1} | {1.75445, 7.55154, 3.} | {{1.75445, 7.55154, 3.}, {1.75445, 7.55154, 3.}} | {1.23276, 12.4364, 2900.} | inf | 882. | 555.223 | {525.228, 587.821} | 37.0496 | 0.201445 | newly | 608. | 410.16 | {382., 449.} | 32.5395 | 0.171659 | Reff | 0.494927 | 0.494559 | {0.493968, 0.495081} | 0.0920116 | ar | 72 941. | 72 351.1 | {72 244., 72 442.} | 0.808777 |
| 42 | R0 | {1.80397, 5.4932, 1.} | {1.81768, 5.68021, 1.} | {{1.81768, 5.68021, 1.}, {1.81768, 5.68021, 1.}} | {0.760022, 3.40431, 0.} | inf | 102. | 246.461 | {227.137, 270.296} | 141.629 | 0.382183 | newly | 82. | 224.63 | {204., 247.} | 173.939 | 0.436364 | Reff | 0.48442 | 0.485476 | {0.485043, 0.485823} | 0.217962 | ar | 73 232. | 73 521.6 | {73 443., 73 596.} | 0.395524 |
| 43 | R0 | {2.83096, 16.7077, 0.1} | {2.93978, 19.9817, 0.} | {{2.93978, 19.9817, 0.}, {2.93978, 19.9817, 0.}} | {3.84376, 19.5959, 100.} | inf | 3887. | 6286.87 | {6257.09, 6313.07} | 61.7409 | 0.208817 | newly | 1377. | 1909.19 | {1855., 1954.} | 38.6485 | 0.141833 | Reff | 0.237546 | 0.176126 | {0.175325, 0.176901} | 25.8561 | ar | 92 740. | 95 187.7 | {95 140., 95 236.} | 2.63929 |
| 44 | R0 | {2.73678, 11.6021, 1.} | {2.8186, 12.0293, 1.} | {{2.8186, 12.0293, 1.}, {2.8186, 12.0293, 1.}} | {2.98974, 3.68172, 0.} | inf | 851. | 1434.6 | {1415.06, 1452.62} | 68.5785 | 0.226781 | newly | 393. | 669.11 | {642., 695.} | 70.257 | 0.230918 | Reff | 0.225237 | 0.215522 | {0.21501, 0.216067} | 4.31343 | ar | 92 003. | 92 741.3 | {92 702., 92 777.} | 0.802419 |
| 45 | R0 | {2.14235, 6.98601, 0.1} | {2.14326, 6.48739, 0.} | {{2.14326, 6.48739, 0.}, {2.14326, 6.48739, 0.}} | {0.0424843, 7.13742, 100.} | inf | 172. | 396.074 | {375.997, 417.859} | 130.276 | 0.361923 | newly | 135. | 308.35 | {284., 331.} | 128.407 | 0.357984 | Reff | 0.36707 | 0.362424 | {0.361952, 0.362846} | 1.26585 | ar | 82 974. | 83 317.2 | {83 272., 83 359.} | 0.413599 |
| 46 | R0 | {2.23409, 9.21289, 0.1} | {2.45478, 11.6771, 3.} | {{2.44675, 11.6528, 3.}, {2.45839, 11.6881, 3.}} | {9.87817, 26.7479, 2900.} | inf | 866. | 2162.2 | {1615.16, 2428.52} | 149.677 | 0.390525 | newly | 520. | 1072.09 | {797., 1223.} | 106.171 | 0.307463 | Reff | 0.341659 | 0.343571 | {0.337457, 0.355822} | 1.98579 | ar | 85 163. | 87 070.3 | {86 289., 87 462.} | 2.23954 |
| 47 | R0 | {2.12258, 8.15265, 0.1} | {2.12684, 8.23415, 1.} | {{2.12684, 8.23415, 1.}, {2.12684, 8.23415, 1.}} | {0.200748, 0.999723, 900.} | inf | 433. | 638.498 | {616.67, 660.935} | 47.4592 | 0.168529 | newly | 280. | 421.11 | {397., 444.} | 50.3964 | 0.176821 | Reff | 0.375293 | 0.371676 | {0.371182, 0.372134} | 0.963841 | ar | 82 690. | 82 896.9 | {82 844., 82 955.} | 0.250248 |
| 48 | R0 | {2.71771, 10.4636, 0.1} | {2.74056, 10.6768, 0.} | {{2.74056, 10.6768, 0.}, {2.74056, 10.6768, 0.}} | {0.840716, 2.03712, 100.} | inf | 924. | 1069.05 | {1049.21, 1086.87} | 15.6986 | 0.0632791 | newly | 498. | 546.44 | {526., 572.} | 9.72691 | 0.040096 | Reff | 0.232147 | 0.230123 | {0.229605, 0.230661} | 0.871697 | ar | 91 716. | 91 915. | {91 872., 91 954.} | 0.216974 |
| 49 | R0 | {2.44369, 6.66477, 0.1} | {2.45956, 6.45922, 4.} | {{2.45956, 6.45922, 4.}, {2.45956, 6.45922, 4.}} | {0.649395, 3.08419, 3900.} | inf | 49. | 36.4903 | {29.0242, 45.0829} | 25.5493 | 0.133813 | newly | 43. | 27.59 | {20., 35.} | 35.8372 | 0.200492 | Reff | 0.273693 | 0.275778 | {0.275562, 0.27594} | 0.761815 | ar | 88 827. | 88 802.2 | {88 791., 88 817.} | 0.028032 |
| 50 | R0 | {2.77383, 17.9775, 0.1} | {2.88523, 18.2127, 0.} | {{2.88523, 18.2127, 0.}, {2.88523, 18.2127, 0.}} | {4.01604, 1.30809, 100.} | inf | 3843. | 6256.56 | {6224.7, 6283.64} | 62.8039 | 0.211662 | newly | 1236. | 2067.01 | {2023., 2110.} | 67.2338 | 0.223261 | Reff | 0.244319 | 0.184494 | {0.183703, 0.185381} | 24.4863 | ar | 92 334. | 94 846.2 | {94 797., 94 908.} | 2.72072 |
